# Topography of the subducting basement throughout the entire Nankai Trough

**DOI:** 10.1038/s41598-025-08846-x

**Published:** 2025-07-30

**Authors:** Kazuya Shiraishi, Yasuyuki Nakamura, Ryuta Arai, Tetsuo No, Yuka Kaiho, Ryo Miura, Ayako Nakanishi, Seiichi Miura, Gou Fujie, Shuichi Kodaira

**Affiliations:** Research Institute for Marine Geodynamics, Japan Agency for Marine- Earth Science and Technology (JAMSTEC), Yokohama, Japan

**Keywords:** Basement topography, Subduction zone, Nankai Trough, Seismic reflection, Earthquake, Geophysics, Structural geology, Seismology

## Abstract

The heterogeneous distributions of large and slow earthquakes in subduction zones are caused by multiple uncertain conditions in the source regions, and the basement topography is considered one of the major controlling factors. We revealed the topography of the subducting basement along the entire Nankai Trough on the basis of seismic reflection profiles compiled from 1997 to 2024. We interpreted the reflection profiles in the time domain to ensure consistency among multiple-generation datasets. The new surface model captured the detailed topography on a scale of several kilometers, over 730 km long and 150 km wide, and down to depths of 15–20 km. The basement topography is characterized by past tectonic activity and is divided into three domains, which affect the present heterogeneity in geological structures and physical properties along the Nankai Trough. While the three domains correspond to megathrust seismogenic zones along the Nankai Trough, the lack of correspondence between the significant topographic relief and the slow earthquake distribution in some areas suggests the need for additional factors controlling spatiotemporal variations in seismicity.

**Supplementary Information:**

The online version contains supplementary material available at 10.1038/s41598-025-08846-x.

## Introduction

The shape of the subducting plate plays an important role in the evolution of geological structures in the overriding plate, and it is considered one of the major factors influencing seismic activity in plate subduction zones^1^. The potential relationships between earthquake activity and topographic features of subducting plates, including undulations with seamounts and ridges, have been documented in many areas in various studies: the Nankai Trough^2–6^, southern Japan Trench^7^, Kuril Trench^8^, Hikurangi margin^9,10^, Cascadia^11^, Costa Rica^12^, Ecuadorian margin^13^, Java margins^14^, and northern Chile^15^. In the Nankai Trough, in addition to historical tsunamigenic earthquakes^16^, recent advancements in seismic observations have revealed slow earthquake occurrence in some specific areas in shallow parts of subduction zones^17,18^. However, regional topographic variations and their relationships with earthquake occurrence are unclear.

**Fig. 1:**
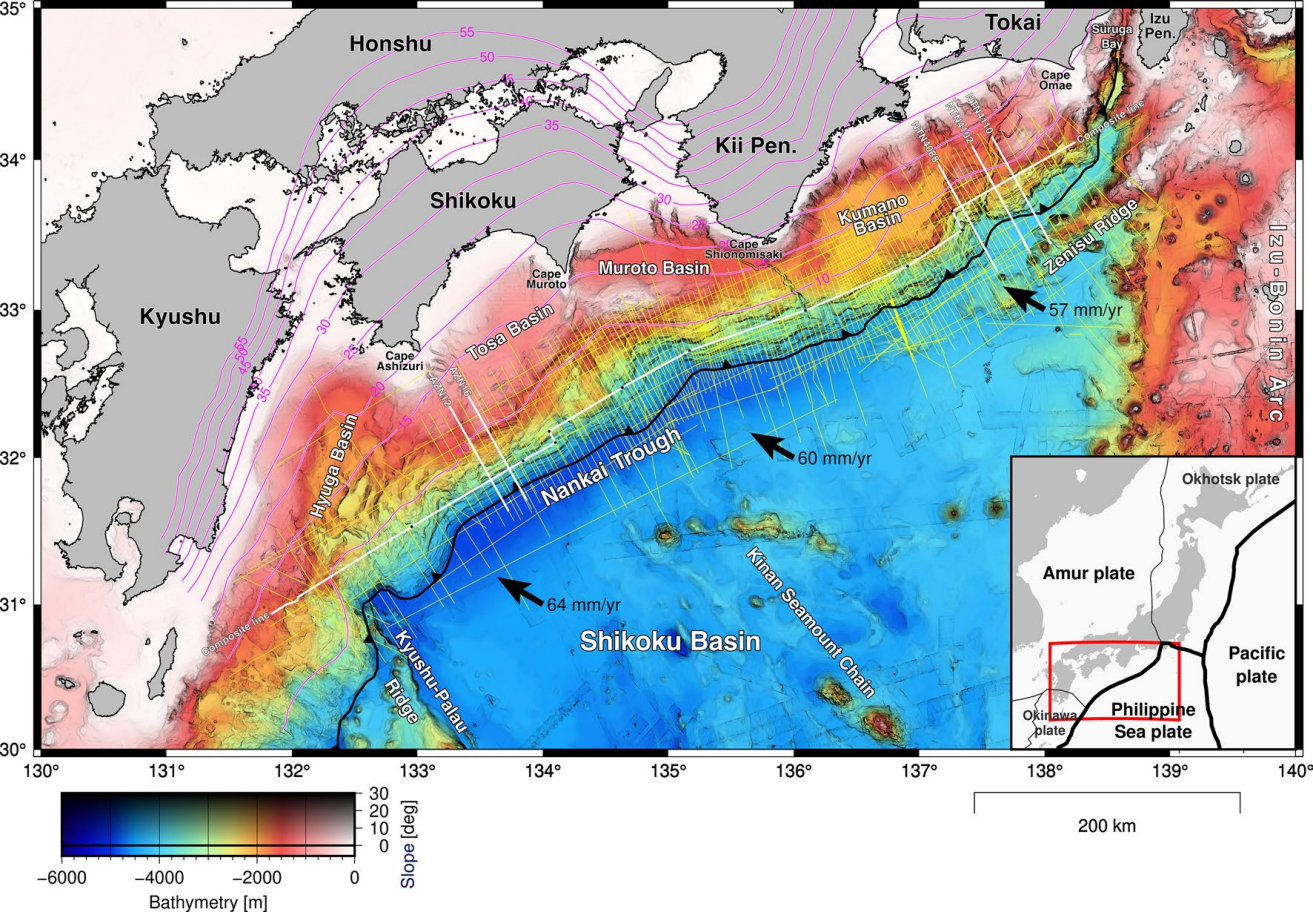
Map showing the location of this study. The yellow lines are survey lines of seismic reflection profiling conducted by JAMSTEC since 1997. The bold white lines indicate the locations of the profiles shown in Fig. 2 (AZN12 and AZN16), Fig. 3 (a composite line), and Fig. 6 (NTN4088, NTN4102, and NTN4110). The black dots on the white line are connection points of survey lines for the composite line. The black arrows represent the direction of movement of the Philippine Sea plate toward the Amur plate according to the NNR-MORVEL56 ^63^. The magenta contours represent the depths of the plate geometry model in a previous study^38^. The bathymetric data are compiled from the bathymetry database of JAMSTEC^64^ and public datasets of Japan Oceanographic Data Center (JODC)^65^ and GEBCO Bathymetric Compilation Group^66^. The map was created using the Generic Mapping Tools (GMT) version 6 ^67^ (https://www.generic-mapping-tools.org/).

The Nankai Trough is located southwest of Japan (Fig. 1), where the Philippine Sea Plate is subducting to the northwest beneath the Amur Plate^19^. Several ridges and seamounts subducting in the Nankai Trough have been documented in previous studies: the northern extension of the Kyushu-Palau Ridge (KPR) at the western end^4,20^, multiple seamounts north of the Kinan Seamount Chain (KSC) in the central part^2,5,21^, and the Paleo Zenisu Ridge (PZR) subparallel to the Zenisu Ridge in the eastern part^3,22^. The KPR was separated from the Izu–Bonin Arc by past seafloor spreading in the Shikoku Basin during 25–15 Ma^23^. The seamounts related to postspreading volcanic activity during 15–7 Ma are located sporadically along the KSC near the spreading center^24^. The Shikoku Basin sediments are composed of thick layers of hemipelagic muds and turbidites with lateral variations in sediment lithology on the rift structures of the Philippine Sea Plate^25,26^. The large volume of sediments caused rapid evolution of the accretionary wedge during plate subduction^27^. Spatial variations in the basement topography and sediment lithology have caused heterogeneous development of geological structures and physical properties within the accretionary wedge^28,29^. While the presence of high pore pressure has been inferred in some parts of underthrust sediments^30–32^, thermogenic gas migrating from deep sediments has been identified in gas samples from shallow sediments related to submarine mud volcanoes^33,34^ and methane hydrate formations^35^. Possible permeable zones for upward fluid migration from near the plate boundary to shallow sediments were deduced via seismic data analysis and were likely developed under the influence of topographic variations in the subducting basement^36,37^.

To elucidate the geological architecture and geophysical conditions, we have conducted many active-source seismic surveys in the Nankai Trough subduction zone. Multichannel seismic (MCS) surveys enable us to image detailed subsurface structures via reflection profiles^3–5,21^. Wide-angle seismic surveys using ocean-bottom seismographs (OBSs) are useful for estimating deeper regional velocity structures than those estimated via MCS surveys^2,29,36,38^. Seismic tomography based on a combination of active-source OBS surveys and earthquake observations is also useful for estimating three-dimensional (3D) velocity structures that are wider and deeper than those estimated via conventional active-source surveys^20^. A regional 3D velocity model and the geometry of the subducting plate were proposed via a compilation of the results of MCS and OBS surveys and local earthquake tomography in southwestern Japan^38^. This regional velocity model was updated via the integration of seismic tomography with numerous datasets of active-source and passive-source data observed from both seafloor and land seismographs^39^. However, the plate surface model was still too smooth to compare topographic features with spatial variations in earthquake activity.

An essential method for understanding the detailed structural characteristics in seismogenic zones is the 3D MCS survey^40–42^. However, since conducting 3D surveys is not easy in the entire region of subduction zones in academic research, a series of dense two-dimensional (2D) MCS surveys is an alternative approach for investigating heterogeneous subsurface structures. The detailed topographic variation with a spatial resolution of several kilometers was revealed by dense 2D profiles^5^, and quantitative analysis of the lateral variation in structural features and their correspondence with slow earthquakes was performed in the middle part of the Nankai Trough^28^.

To clarify the crustal structures throughout the entire Nankai Trough, we conducted an intensive multiyear seismic survey campaign with approximately one-month cruises each year since 2018. In addition to legacy datasets before 2018, dense 2D seismic surveys covered most of the Nankai Trough subduction zone at intervals of 4–8 km between survey lines. In this study, to investigate the topography of the subducting basement along the entire Nankai Trough, we compiled seismic reflection profiles collected from 1997 to 2024. Then, we interpreted reflections at the boundary between the sediments and the basement on the subducting plate and generated a new surface model of the detailed basement topography. In this manuscript, we discuss the topographic features and potential associations with earthquake activity.

## Results and discussion

### Seismic reflection profiling of structural variations

**Fig. 2:**
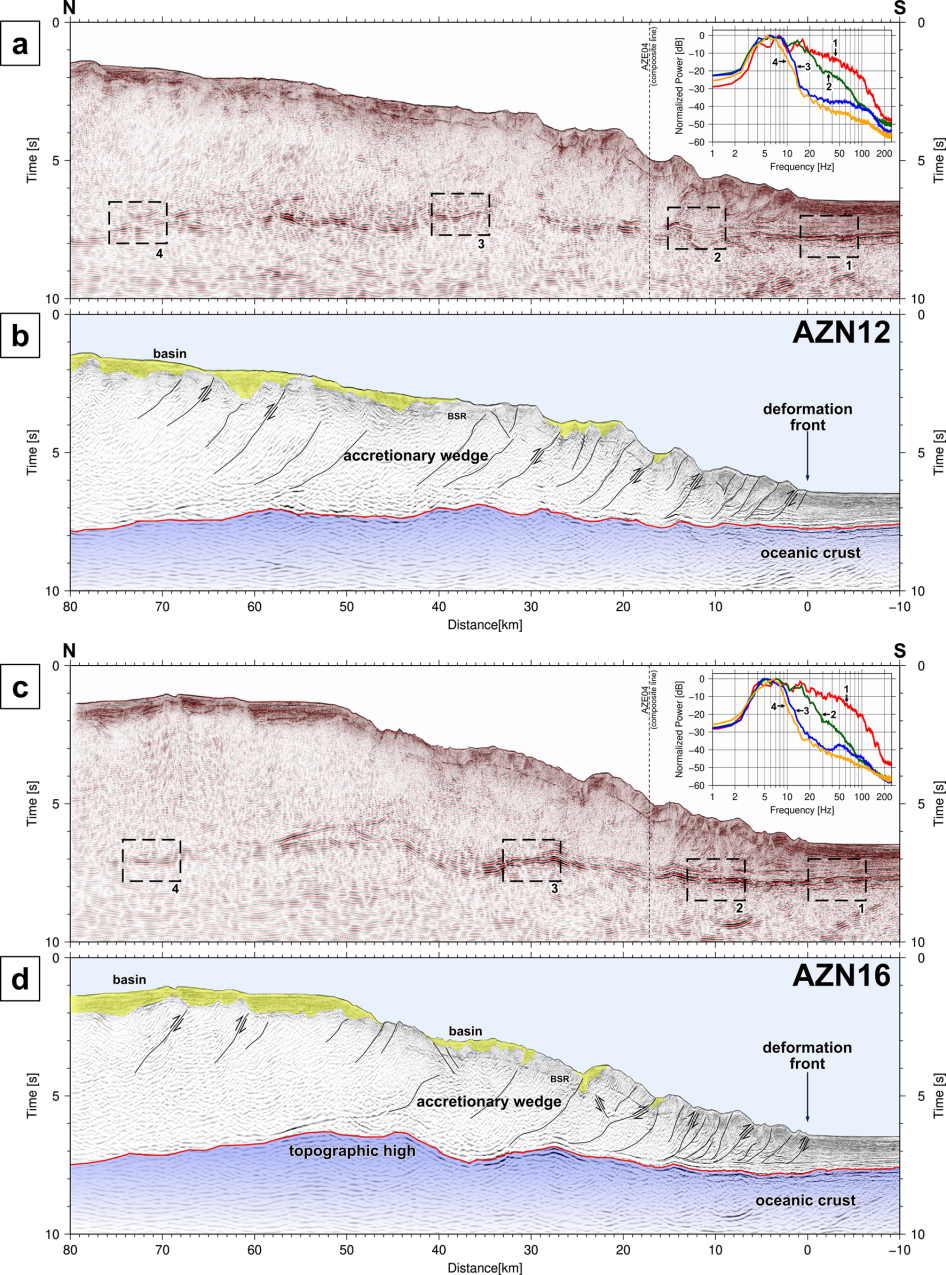
Seismic reflection profiles along the survey lines of AZN12 and AZN16. (**a**) Uninterpreted and (**b**) interpreted cross-sections of AZN12, (**c**) uninterpreted and (**d**) interpreted cross-sections of AZN16. The subpanels in (a) and (c) show the amplitude spectra at the boxed areas in (a) and (c), respectively. The black lines in (b) and (d) are possible faults. The red lines represent the basement surface. The yellow and blue translucent areas represent sedimentary basins and the basement, respectively.

**Fig. 3:**
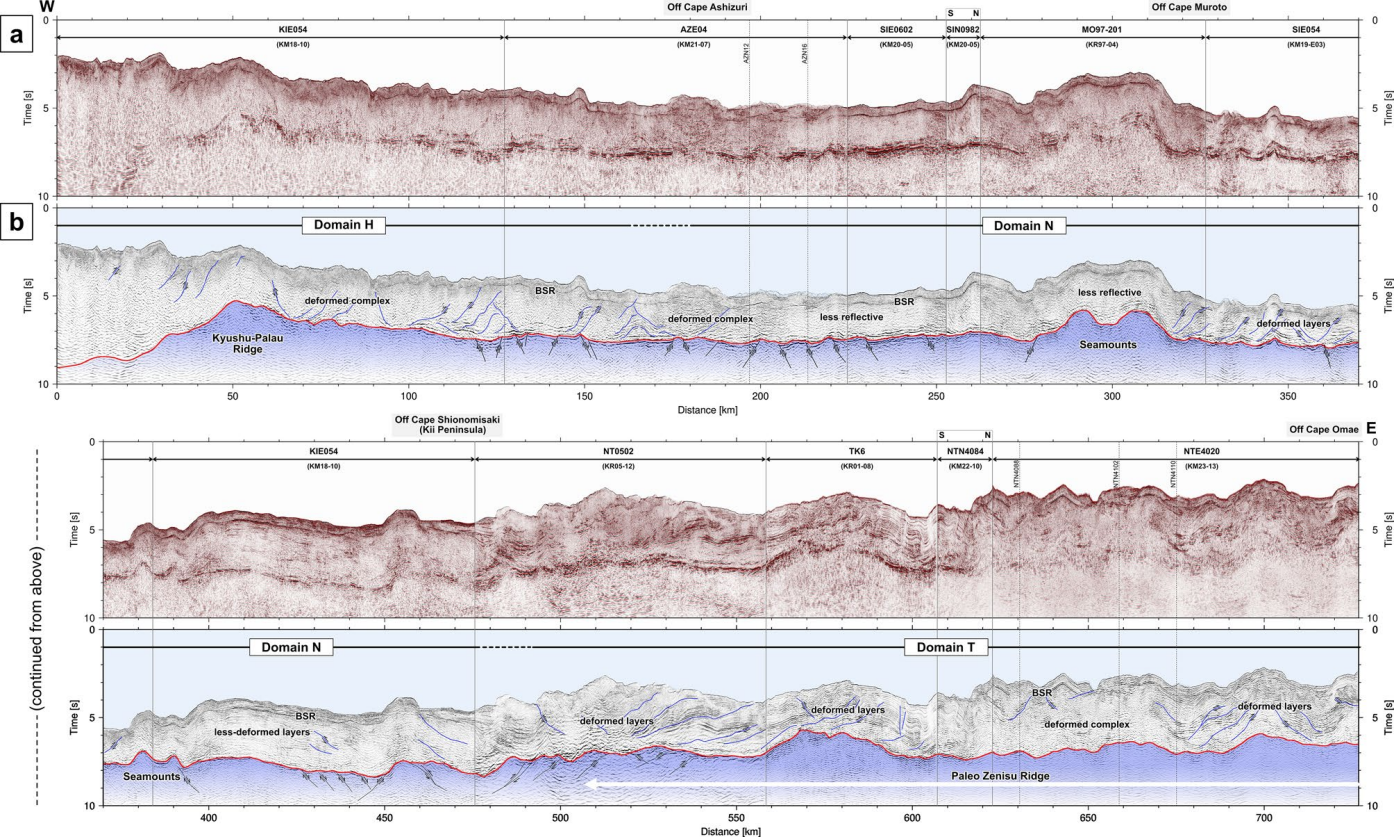
Seismic reflection profile along the composite line. (**a**) Uninterpreted and (**b**) interpreted cross-sections. The blue translucent part shows the basement, and the black lines represent possible faults.

Dense reflection surveys revealed subsurface geological structures along the Nankai Trough. In the reflection profiles along the seismic lines off Cape Ashizuri (Fig. 2), the basement reflections are traceable from 1.5 s at the southern end to 6 ~ 6.5 s at the northern end. The thickness of the accretionary wedge generally increases with increasing distance from the deformation front. While the basement in the AZN12 profile has a relatively flat surface with minor undulation (Fig. 2a-b), the basement reflection in the AZN16 profile has a mound shape approximately 50 km from the deformation front (Fig. 2c-d). Assuming that the velocity structure in the accretionary wedge is similar between the two survey lines only 16 km apart, the thickness of the accretionary wedge is approximately the same in both profiles, and the depth of the basement in the AZN16 profile should be shallower than that in the AZN12 profile in the range of 50–80 km from the deformation front, as well as the depth of the seafloor. The reflection profile along the composite line connecting multiple survey lines represents lateral variations in the topography and deformation structures from the western end to the eastern end along the Nankai Trough (Fig. 3). The basement topography represents significant variation along the profile, with features including ridges and seamounts. While normal faults are widely distributed in the middle part, reverse faults exist within the underlying basement southeast of the Kii Peninsula. The reflection features within the accretionary wedge change spatially: patterns of layers that are less deformed or deformed by faults and folds, and patterns that are less reflective or scattered, possibly due to deformed complexes. Intermittent reflections subparallel to the seafloor are known as bottom simulating reflectors (BSRs), which imply the existence of gas hydrates^43^. The structural deformations observed in the orthogonal survey lines (Figs. 2 and 3) suggest complex 3D effects on the development of the accretionary wedge because of oblique subduction to the trench with undulations of the basement.

**Fig. 4:**
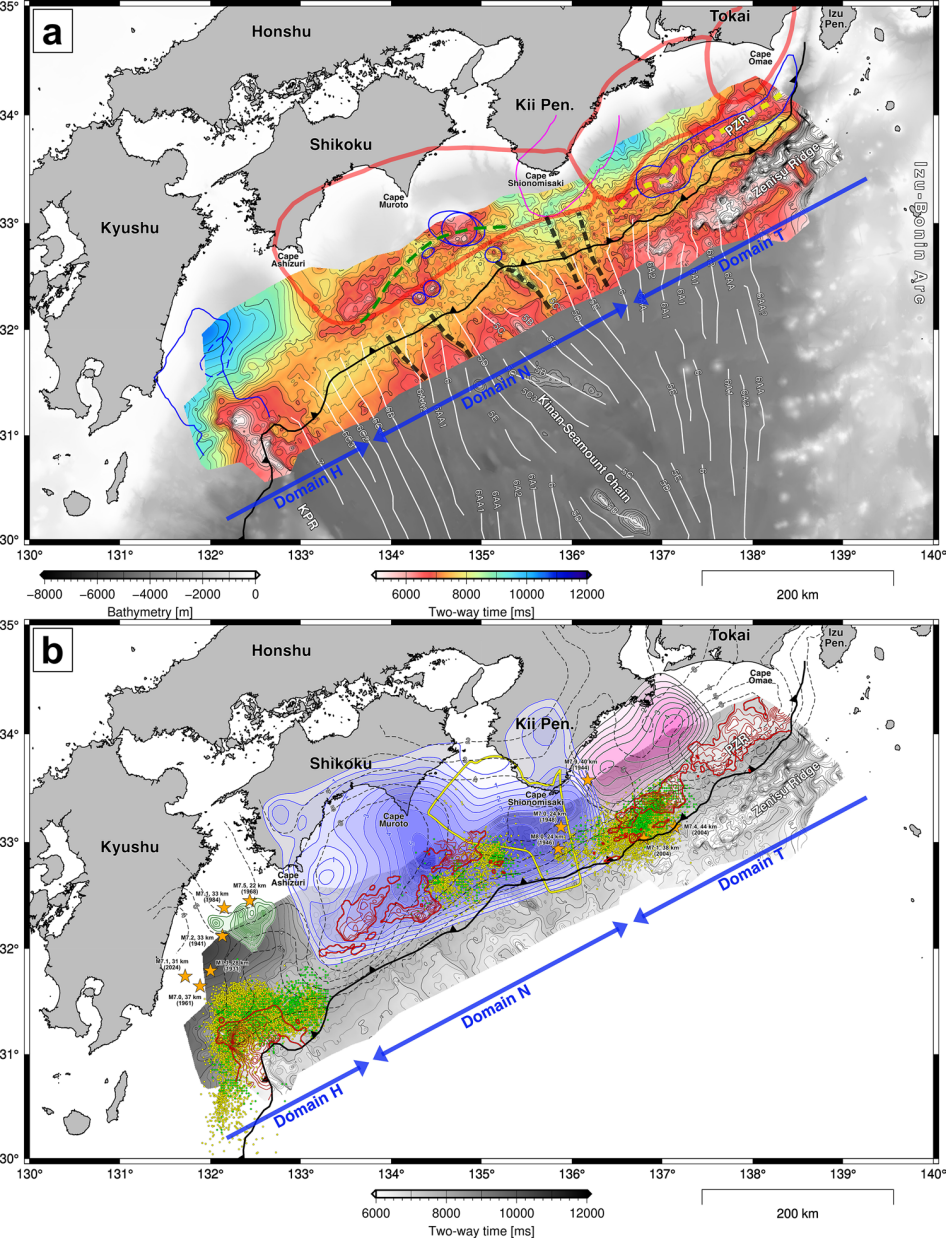
Surface topography of the subducting basement. (**a**) The basement topography can be divided into three domains: H, N, and T. The black dashed lines represent linear depressions in the north–south direction. The green dashed line shows an arch-shaped valley feature. The regions outlined by the red bold lines are megathrust seismogenic zones: Nankai, Tonankai, and Tokai from west to east^17^. The white lines represent the magnetic lineaments on the Philippine Sea plate^44^. The circles and curves with blue lines indicate the locations of seamounts documented in previous studies^2,5,21,42^ or ridges^3,20,36^ on the subducting plate. The magenta line shows the area of inferred subsurface distribution of plutonic rocks^46^. (**b**) Spatial relationship between the topography of the basement and seismic activity. The red curves show the contours of the basement surface shallower than 7,000 ms in two-way traveltime every 250 ms. The orange-colored stars denote epicenters of earthquakes of magnitude M ≥ 7. The pink, blue, and green translucent areas represent the slip regions (unit: m) of the Tonankai earthquake in 1944^51^, the Nankai earthquake in 1946^49^, and the Hyuganada earthquake in 1968 ^68^, respectively. The area outlined by the yellow line is a seismically active region of microearthquakes in the four years of observations from 2003 to 2007 ^50^. The yellow and green dots are epicenters of tectonic tremors^69–72^ and very low-frequency earthquakes^73–78^, respectively. The black dashed lines represent the slip deficit rate (unit: cm/yr)^56^. The maps were created using the Generic Mapping Tools (GMT) version 6 ^67^ (https://www.generic-mapping-tools.org/).

**Fig. 5:**
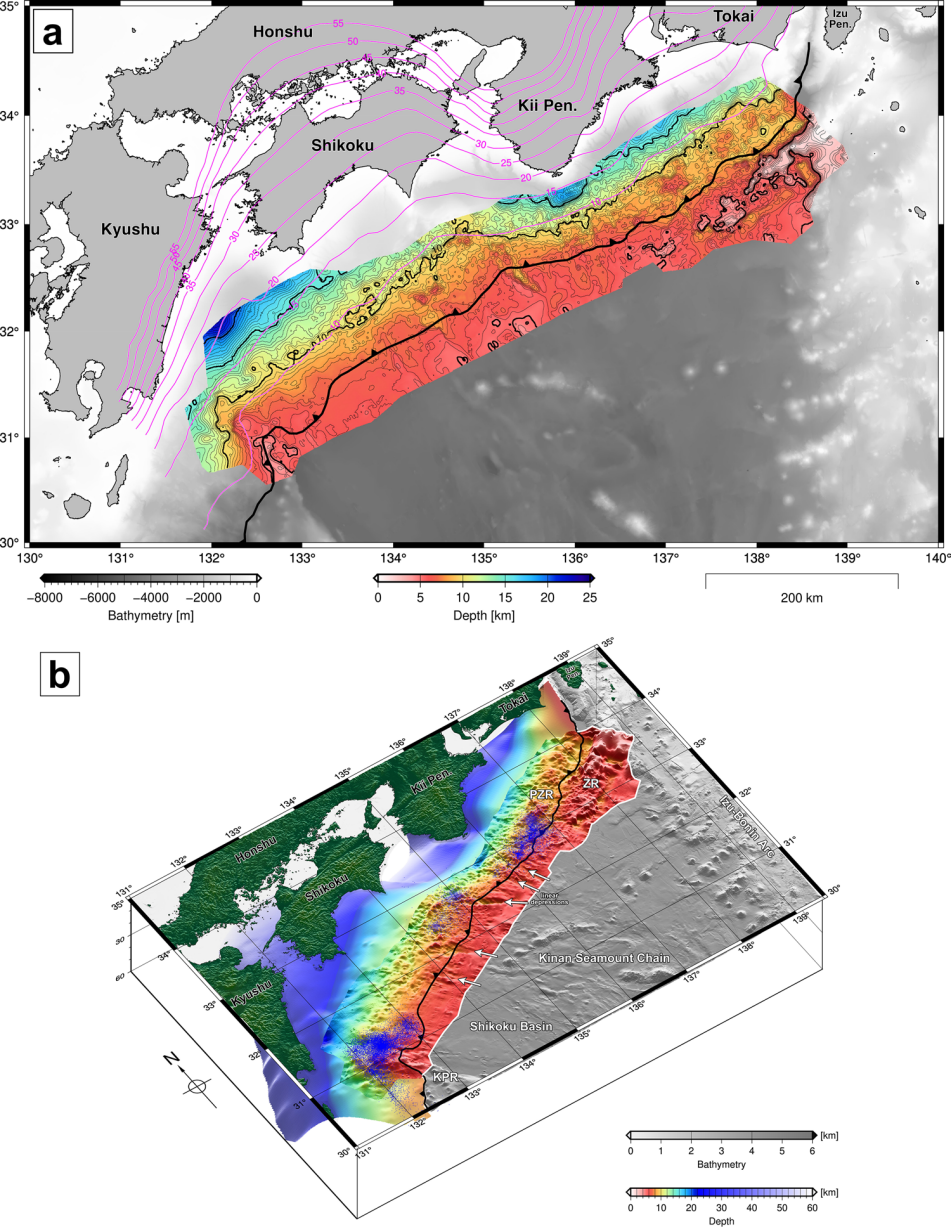
The basement surface in the depth domain. (**a**) Surface topography of the subducting basement converted from time to depth (color map) using a regional 3D velocity model^39^ and comparison with an existing plate geometry model^38^. (**b**) A perspective view of the time-to-depth converted surface (this study) superimposed on the plate geometry model^38^. The black line shows the deformation front of the accretionary wedge. The white arrows represent linear depressions. The blue dots are hypocenters of slow earthquakes^69–78^. KPR: Kyushu-Palau Ridge; PZR: Paleo Zenisu Ridge; ZR: Zenisu Ridge. The maps were created using the Generic Mapping Tools (GMT) version 6^67^ (https://www.generic-mapping-tools.org/).

The generated surface, which is based on the interpretation along 247 lines (Supplementary Fig. S1), represents the topography of the basement over a length of 730 km and a width of 150 km along the entire Nankai Trough subduction zone (Fig. 4 and Supplementary Fig. S2). Continuous seismic surveys with dense survey lines that have been conducted for more than two decades have enabled us to capture detailed topographic features at the scale of several kilometers (Supplementary Fig. S3). We identified three major regions with significant topographic highs on the north side of the deformation front. The region of the western topographic high off Kyushu is located on the northern extension of the KPR, forming a north–south trending ridge at the west edge of the Shikoku Basin. The eastern region of topographic highs south of Tokai consists of multiple ridges aligned subparallel to the Zenisu Ridge. The region of topographic highs in the middle consists of several areas of topographic relief with arc-shaped depressions (Fig. 4a, green dashed line). Additionally, we identified several linear depressions in the north–south direction crossing the deformation front (Fig. 4a, black dashed lines). The deepest part is located off the coast of Kyushu, and relatively deep parts exist around the Kii Peninsula. The time-to-depth domain-converted surface based on a regional 3D velocity model^39^ superimposed on the existing plate model^38^ is shown in Fig. 5. While the general trend of depth changed to deepening in the subduction direction after the domain conversion, local topographic features remained at the surface down to depths of 15–20 km.

## Along-trough topographic variations

The basement surface can be divided into three domains on the basis of topographic features (Fig. 4a, blue arrows): western domain H (Hyuganada), middle domain N (Nankai), and eastern domain T (Tokai). In western domain H, the significant topographic high is the northern extension of the KPR at the western end of the Shikoku Basin. The deep portion of the subducting KPR toward Kyushu was inferred via seismic tomography on the basis of active-source and passive-source data^20^ (Fig. 4a, blue outlined area), and a local topographic high on the subducted KPR was noted on the basis of geomagnetic anomalies^36^ (Fig. 4a, blue dashed outlined area). However, because the subducting plate steeply deepens toward Kyushu^38^, detecting these deep structures via conventional active-source seismic surveys is difficult, and there is insufficient coverage due to sparse survey lines in this region (Supplementary Fig. S1).

In middle domain N, the north–south oriented linear depressions seem to correspond to the lineaments representing the geomagnetic anomaly (Fig. 4a)^44^. These linear depressions suggest rift structures caused by normal faulting in an extensional regime resulting from past seafloor spreading in the Shikoku Basin^24^, and corresponding depressions have been documented in previous studies on the basis of seismic reflection profiles^25^. On the north side of the deformation front, in addition to the topographic highs associated with the subducted seamounts inferred by previous seismic surveys off Cape Muroto (Fig. 4a, blue circles)^2,5,21,42^, we identified a broad topographic high part off Cape Ashizuri. The subducted seamounts or ridges identified in the middle of domain N are located north of the linear depressions. These topographic highs generally correspond to high gravity anomalies, suggesting the presence of denser rock bodies than in the surrounding area (Supplementary Fig. S4). There are several terrestrial outcrops of middle Miocene plutonic intrusions on Cape Ashizuri, Cape Muroto, and the southern Kii Peninsula^45^. The presence of a large subsurface pluton was inferred from gravity and seismic velocity anomalies offshore of the Kii Peninsula^46,47^. These plutons, which may cause high-gravity anomalies, are located north of linear depressions. In the middle of the Shikoku Basin, several high-gravity anomalies correspond to seamounts in the KSC (Supplementary Fig. S4). Consequently, the association between the topographic highs and the high-gravity anomalies in domain N implies that the subduction of seamounts or ridges developed during past igneous activity at the rifts in the Shikoku Basin^23,24^. The arc-shaped valley was noted as a possible apparent feature caused by the existence of low velocities^5^, which may be relevant to fluid-rich fracture zones in the overriding accretionary wedge that have been affected by ridge subduction^37^.

In eastern domain T, the high topographic relief identified on the north side is associated with the subducted ridges of the PZR that have been recognized via magnetic anomalies^48^, seismic velocities^22^, and reflection profiles^3^. The topographic high parts correspond to the high gravity anomalies extending westward from the Izu–Bonin Arc, as well as the Zenisu Ridge (Supplementary Fig. S4). As a result of the additional dense seismic profiles, we revealed the detailed geometry of the topographic relief in the PZR. Several isolated ridges are distributed on the elongated regional topographic high farther west (Fig. 4a, yellow dashed line) than in the area that was previously depicted (Fig. 4a, blue outlined area).

## Potential associations with earthquake activity

The three domains, which are based on the topographic features of the basement, correspond approximately to segments of the megathrust seismogenic zone along the Nankai Trough (Fig. 4a). The western domain H is the area where large earthquakes of magnitude M ≥ 7.0 have occurred repeatedly in the deep portion of the subducting plate east of Kyushu (Fig. 4b). The middle domain N corresponds to the Nankai earthquake segment. The slip distribution of the Nankai earthquake in 1946 ^49^ appeared along the topographic relief south of Shikoku. The deep portion on the eastern side of domain N corresponds to an active region of crustal earthquakes determined from the 4-year seafloor seismic observation^50^ (Fig. 4b, area outlined by the yellow line). The eastern domain T corresponds to the Tonankai and Tokai segments (Fig. 4a). The major slip of the Tonankai earthquake in 1944 ^51^ was distributed southeast of the Kii Peninsula and attenuated as it approached the topographic highs of the PZR (Fig. 4b). Subducted seamounts or ridges have been considered barriers to rupture propagation during earthquakes^2,3^. Studies of analog and digital geological modeling, however, have suggested that the subduction of multiple seamounts or ridges may generate smooth detachments over rough undulations of the basement^52,53^, such as an actual smooth decollement^28^, which could be a potential area of coseismic slip. Because fault geometry is one of the major factors affecting slip tendencies^54,55^, in some situations, wide areas could slip beyond ridges during large tsunamigenic earthquakes, such as the Nankai earthquake^49^.

**Fig. 6:**
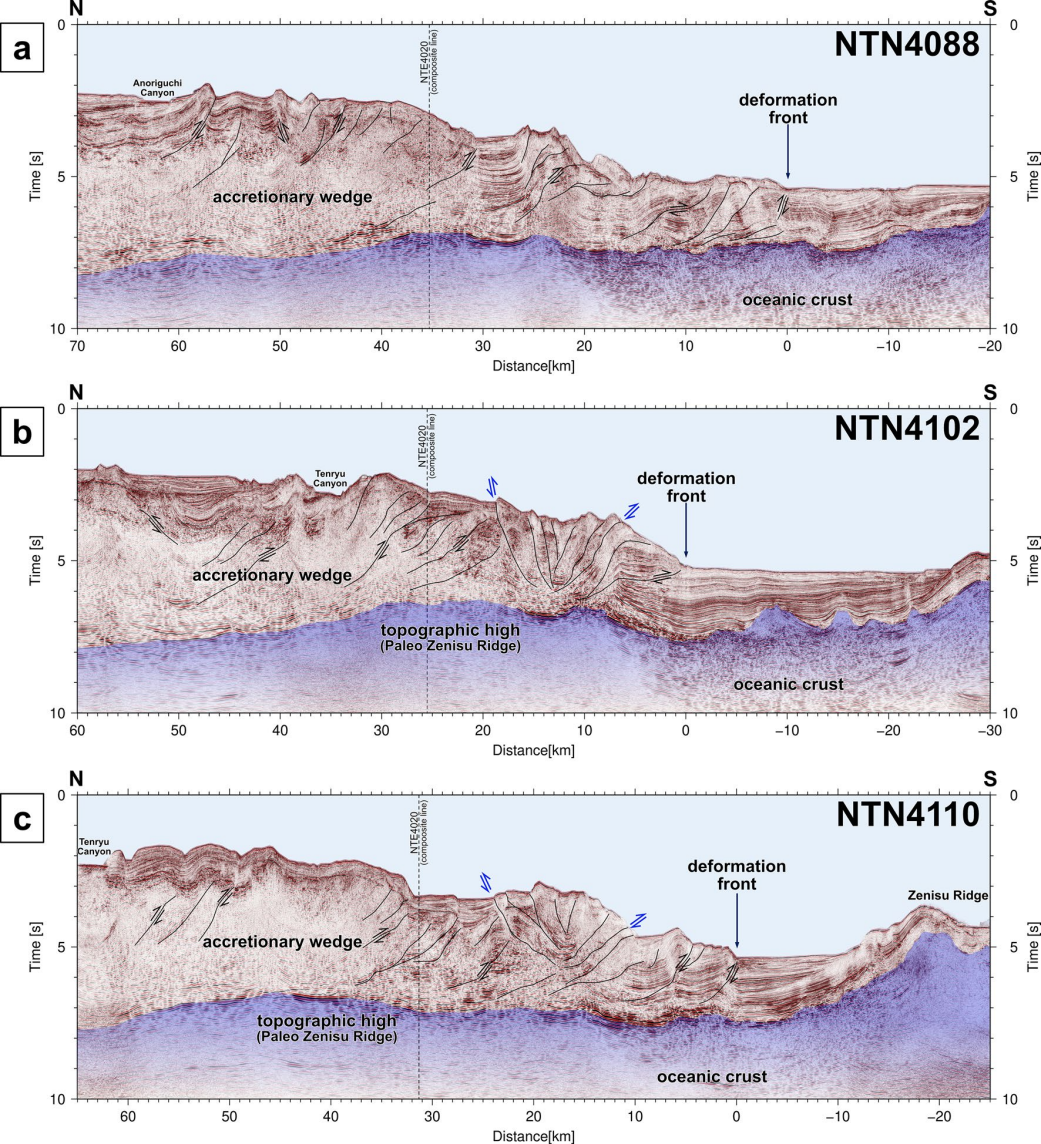
Seismic reflection profiles off Tokai. Sections along survey lines (**a**) NTN4088, (**b**) NTN4102, and (**c**) NTN4110. The blue translucent parts represent the basement. The black lines represent interpreted reverse faults.

Most of the slow earthquakes that have occurred over the past several decades (Fig. 4b, green and yellow dots) seem to be distributed near the topographic reliefs: around the KPR-related high in domain H, the trough side of reliefs off Cape Muroto in domain N, and southeast of the Kii Peninsula in domain T. In contrast, few slow earthquakes have been observed, even around the high topographic reliefs in the western part of domain N and eastern part of domain H, where high slip deficit rates were estimated from onshore and offshore geodetic data^56,57^ (Fig. 4b, dashed line contours). The broader ridges off the Cape Ashizuri and Tokai regions might have caused strong interplate coupling with the low activity of slow earthquakes. Especially in the eastern part of domain T, the subparallel presence of the subducted ridges of the PZR and the Zenisu Ridge may have generated different situations of internal deformation and physical conditions in the accretionary wedge compared with other parts along the Nankai Trough. While major landward-dipping thrusts with minor backthrusts are common in most of the Nankai Trough (Figs. 2 and 6a), both major landward- and trenchward-dipping thrusts are identified in the eastern part of domain T (Fig. 6b-c). As suggested in a previous study via analog model experiments^52^, the subducting broad ridge of the PZR may have caused specific deformations in the eastern part of domain T. The subduction of seamounts and ridges can cause spatiotemporal changes in the internal stress state and pore fluid pressure in overriding sediments^52,53,58^.

The lack of correspondence between the significant topographic relief and the slow earthquake distribution suggests the need for other factors controlling spatiotemporal variations in slip behaviors^59^. In addition to the effects of seamount subduction on the mechanical and hydraulic properties^58^, along-trough variations in lithology and stratigraphy in the input sediments^26^ on the topographic reliefs of the basement with multiple seamounts may affect geological structure evolution with variable deformations and physical conditions in the overriding sediments^60^, which could cause local heterogeneity influencing the slow earthquake distribution. Seismological monitoring suggests temporal changes in pore fluid pressure due to fluid migration in the sediments, which may influence the occurrence of slow earthquakes^61^. Investigations based on the high-density seismic profiles will elucidate the local heterogeneity of internal structures and possible fluid migration paths in the overriding sediments and their relationship to the basement topography. Further geological modeling combining multiple scales of subducting seamounts/ridges and physical properties reflecting lithological variations may help in understanding heterogeneous geological evolution and seismic activity.

## Methods

### Seismic reflection data

To investigate the geological architecture and physical properties in the Nankai Trough subduction zone, the Japan Agency for Marine-Earth Science and Technology (JAMSTEC) has continuously conducted seismic surveys (Fig. 1 and Supplementary Table S1). In this study, we used the seismic reflection profiles of legacy datasets along sparsely distributed survey lines from 1997 to 2017 and new datasets acquired along densely distributed survey lines during a series of MCS survey cruises from 2018 to 2024. Except for the western edge, the MCS survey lines cover most of the Nankai Trough at intervals of 4–8 km in the direction orthogonal to the trough axis and 10–25 km parallel to the trough axis.

The new data acquired from 2018 to 2023 and some of the legacy data before 2018 were processed by recent standard processing workflows, which include deghosting, debubbling, noise reduction techniques, and multiple reflection attenuation by surface-related multiple elimination and parabolic radon filtering. Then, prestack time migration (PSTM) was applied to generate reflection profiles of the vertical axis in time. These processes were conducted by geophysical contractors, DUG Technology, and JGI, Inc. Legacy datasets before 2018 and the newest dataset acquired in 2024 were processed in-house by JAMSTEC via fundamental methods of preprocessing and common-midpoint (CMP) stacking followed by poststack time migration.

### Basement surface model

In this study, we interpreted reflections at the basement surface in two-way traveltime seismic reflection profiles, as shown in Figs. 2 and 3. By compiling the reflection profiles with vertical axes in time, we consistently interpreted many profiles derived from different data acquisitions (Supplementary Table S1). For instance, we confirmed that the key reflection surfaces (i.e., the seafloor and basement) are continuous across the different profiles along a composite line (Fig. 3). We used the commercial software Petrel (released by SLB (formerly Schlumberger)) for seismic interpretation. The distributions of the interpreted traveltimes of the reflections at the basement surface for the 247 survey lines of the 25 cruises are shown in Supplementary Fig. S1. When the reflections were obscure or there were similar survey lines in the vicinity, we did not always interpret them at every survey line.

The vertical resolution depends on the frequency of the seismic wave. Because the high-frequency component decays as the seismic wave propagates through the accretionary wedge (see frequency analysis panels in Fig. 2a and c), the vertical resolution at the basement surface generally decreases with increasing distance from the deformation front. The wavelength is convertible from the frequency with certain velocity values; for example, the wavelength at a frequency of 10 Hz is 30 m if the velocity is 3000 m/s and 50 m if the velocity is 5000 m/s, and the wavelength at a frequency of 20 Hz is half for each.

Next, we generated a basement surface model by interpolating the interpreted reflection boundaries on the profiles. We generated a surface model with 500 m × 500 m grids via the minimum curvature interpolation method implemented in the seismic interpretation software. The minimum distances between the grid points on the interpolated surface and the nearest survey line for each point (Supplementary Fig. S3) represent the substitutional spatial resolution for detectable structural features, which is less than a few kilometers in most parts of the middle to eastern parts. In the western part, where the density of survey lines is relatively low, the detectable resolution decreases to 8–12 km or a maximum of ~ 20 km in some locations.

## Time-to-depth conversion

We created a basement surface model of reflection traveltimes on the basis of consistent interpretations among the new and legacy data. Advanced seismic imaging techniques, such as prestack depth migration, are available for direct investigations of depth domain profiles^5,37^. However, determining the actual depth of reflections is generally not easy because of uncertainty in velocity estimation with the MCS data, especially at depths greater than the length of the streamer cable. To overcome the problems of uncertain velocity determination, wide-angle seismic data are essential for estimating reliable velocities deeper than those derived from MCS reflection data^36,62^. Here, we attempt to convert the surface from the time domain to the depth domain on the basis of an existing regional 3D velocity model^39^ with interpretation software. The time-to-depth domain conversion is based on the relationship between vertical depth and time, which involves velocity variation with depth from the datum level (i.e., mean sea level) to the basement surface. The depths of the domain-converted surface are mostly comparable with the depths of the existing plate model^38^ (Fig. 5).

## Electronic supplementary material

Below is the link to the electronic supplementary material.

Supplementary Material 1

## Data Availability

The seismic data and results in this study are available upon reasonable request from the JAMSTEC seismic survey database (10.17596/0002069).
