# Supplementary material for: Topography of the subducting basement throughout the entire Nankai Trough
Source: Sci Rep. 2025 Jul 30;15:25530. doi: 10.1038/s41598-025-08846-x (PMC12311142; doi:10.1038/s41598-025-08846-x)
Supplement: Supplementary file 1 — Supplementary Material 1 [file 41598_2025_8846_MOESM1_ESM.pdf]

Supplementary information for

Topography of the subducting basement throughout the entire Nankai Trough

Kazuya Shiraishi [a, *], Yasuyuki Nakamura [a], Ryuta Arai [a], Tetsuo No [a], Yuka Kaiho [a], Ryo Miura [a], Ayako Nakanishi [a], Seiichi Miura [a], Gou Fujie [a], and Shuichi Kodaira [a]

[a] Research Institute for Marine Geodynamics, Japan Agency for Marine-Earth Science and Technology (JAMSTEC), Yokohama, Japan

[*] Corresponding author
Kazuya Shiraishi
Research Institute for Marine Geodynamics, Japan Agency for Marine-Earth Science and Technology, 3173-25 Showa-machi, Kanazawa-ku, Yokohama, Kanagawa, 236-0001, Japan
Email address: kshiraishi@jamstec.go.jp

Contents of this file

Supplementary Table S1
Supplementary Figures S1–S4

Supplementary Table S1.

Basic specifications of the seismic reflection surveys used in this study.

Cruise name	Year.Month	Vessel name	Airgun total volume (cu.in.)	Shot interval (m)	Airgun depth (m)	Streamer length (km)	Channel interval (m)	No. of channels	Streamer Depth (m)	Sampling Interval (ms)	Record length (s)
KM24-12	2024.10	Kaimei	10,600	50	10	5.5	12.5	444	25	2	14
KM23-13	2023.10	Kaimei	10,600	50	10	5.5/4.5	12.5/3.125	444/1440	25	2	14
KM22-10	2022.08	Kaimei	10,600	50	10	5.5	12.5	444	25	2	14
KM21-07	2021.09	Kaimei	10,600	50	10	5.5	12.5	444	25	2	14/16
KM20-05	2020.08	Kaimei	10,600	50	10	5.5	12.5	444	25	2	14
KR19E-03	2019.12	Kairei	7,800	50	10	5.5	12.5	444	25	2	14
KM18-10	2018.11	Kaimei	5,300	50	10	4.5	3.125	1440	25	2	14
YK16-13	2016.06	Yokosuka	380	37.5	3	1.2	6.25	192	4	1	12
KY14-07	2014.05	Kaiyo	380	37.5	3	1.2	6.25	192	4	1	12
KY13-11	2013.08	Kaiyo	380	37.5	3	1.2	6.25	192	4	1	12
KR12-12	2012.06	Kairei	7,400	50/200	10	5.5	12.5	444	12	2	12/14/15
MR12-01	2012.05	Mirai	240	37.5	3	1.2	6.25	192	4	1	12
KY12-02	2012.02	Kaiyo	320	37.5	3	1.2	6.25	192	4	1	12
KR11-09	2011.09	Kairei	7,800	50/200	10	5.5	12.5	444	12	2	15
KR10-11	2010.10	Kairei	7,800	200	10	5.5	12.5	444	12	2	15
KR05-12	2005.08	Kairei	12,000	50	10	5.1	25	204	15	4	14
ODKM03	2003.01	Polar Princess	4,240	50	6	6	12.5	480	10	2	11
KR02-11	2002.08	Kairei	12,000	50	10	5.1	25	156–204	20	4	13.5
KR01-14	2001.11	Kairei	12,000	50	10	3.9	25	156	30/15	4	13.5
KR01-08	2001.05	Kairei	12,000	50	10	3.9	25	156	15	4	13.5
KR99-04	1999.05	Kairei	12,000	50	10	3.9	25	156	15	4	13.5
KR98-10	1998.10	Kairei	4,000	50	10	3	25	120	15	4	13.5
KR98-06	1998.06	Kairei	4,000	50	10	3	25	120	15	4	13.5
KR97-04	1997.06	Kairei	3,080	50	10	3	25	120	10	4	13.5
KR97-02	1997.05	Kairei	3,080	50	10	3	25	120	15	4	13.5

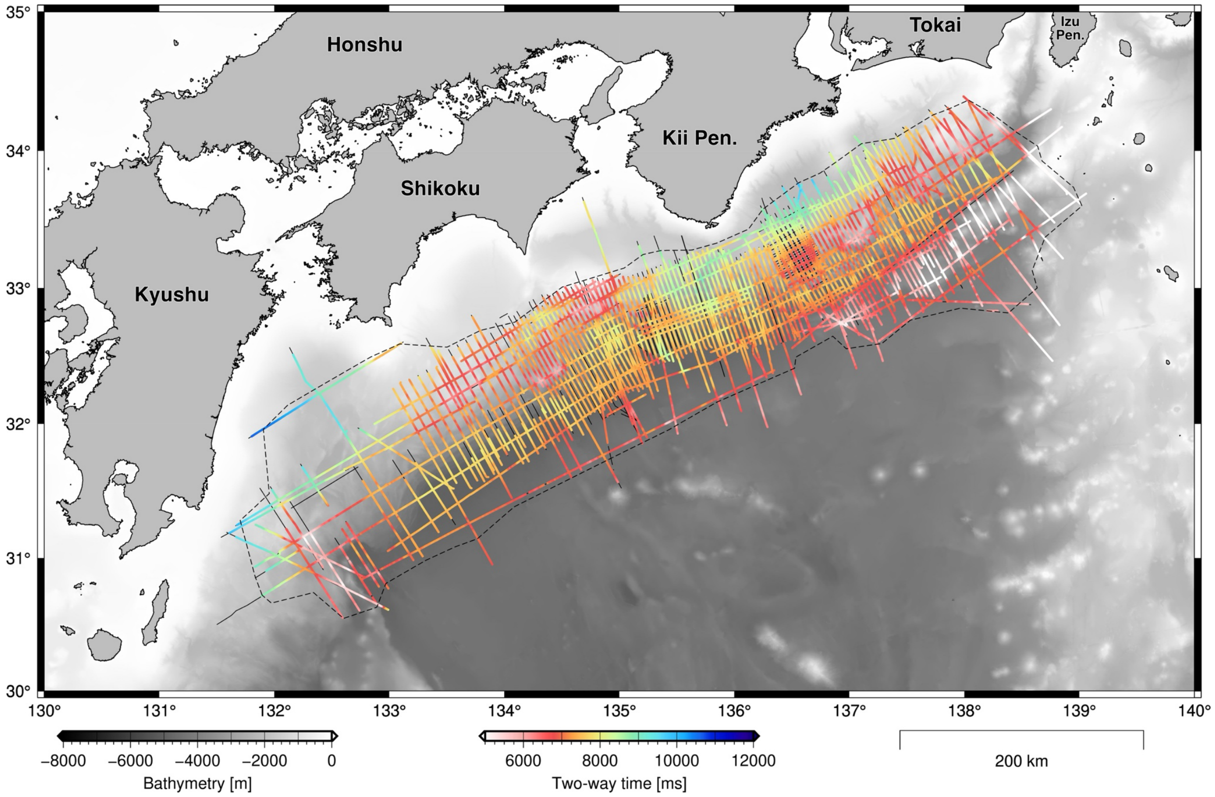

Supplementary Figure S1. Distribution of interpreted survey lines. The colored lines represent two-way traveltime interpreted reflections at the top of the subducting basement. The black dashed line shows the outline of the interpolated surface.

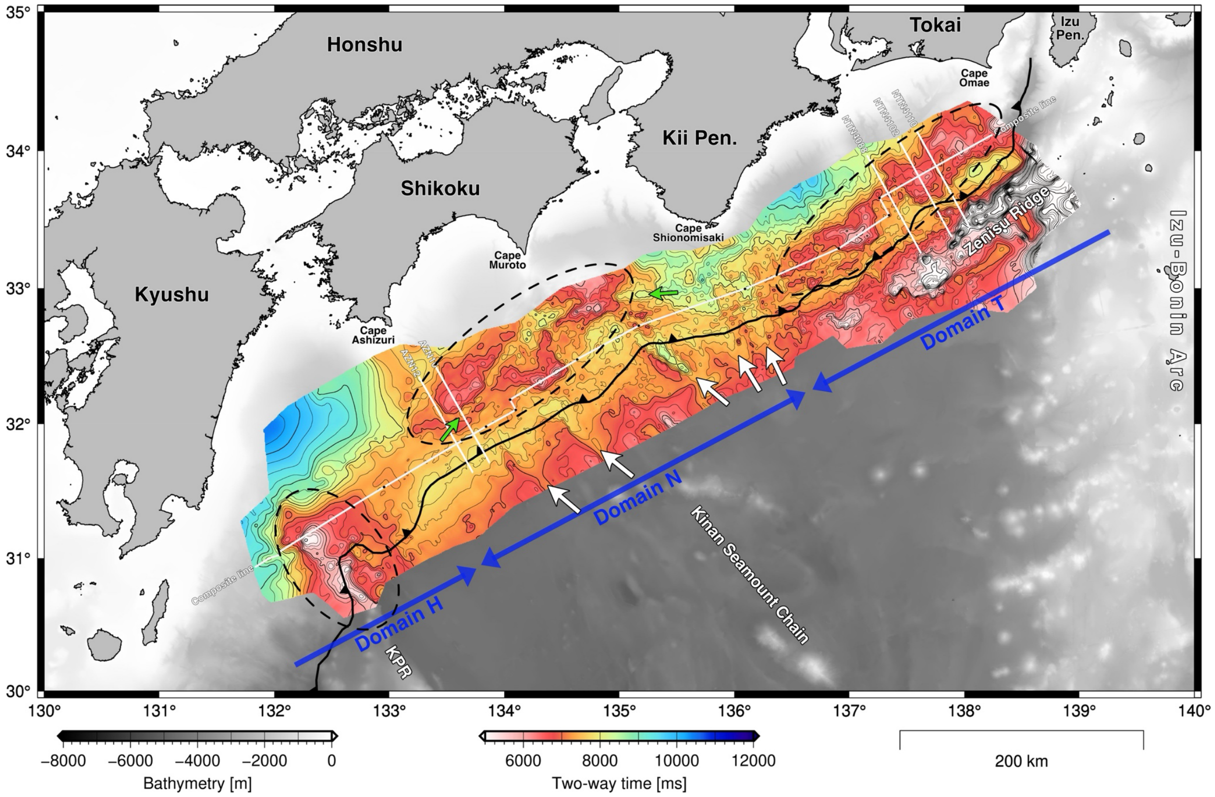

Supplementary Figure S2. Interpolated surface topography of the subducting basement. The black dashed line ellipses indicate regional topographic high parts. The white arrows indicate linear depressions in the north–south direction. The green arrows indicate the arc-shaped depressions. The white lines indicate the locations of the seismic reflection profiles shown in Figs. 2, 3, and 6.

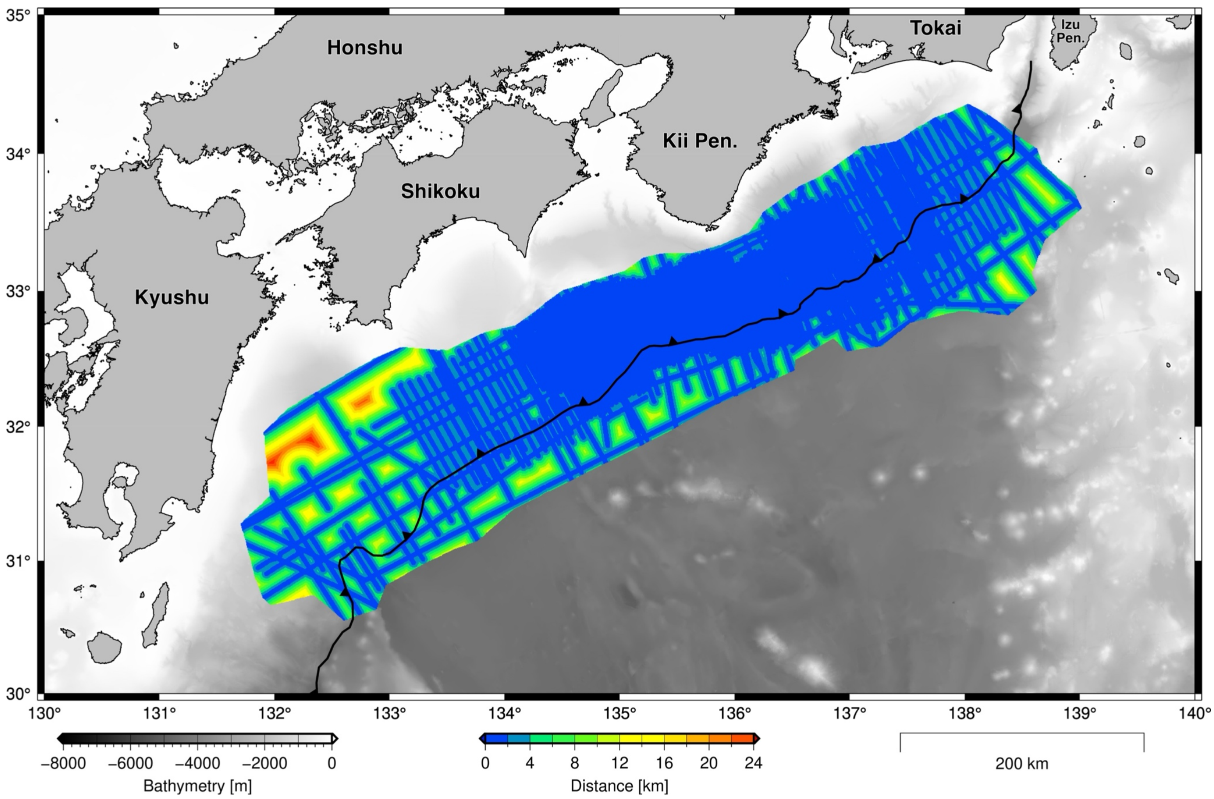

Supplementary Figure S3. Distribution of the minimum distance between each point on the interpolated basement surface and the nearest survey line.

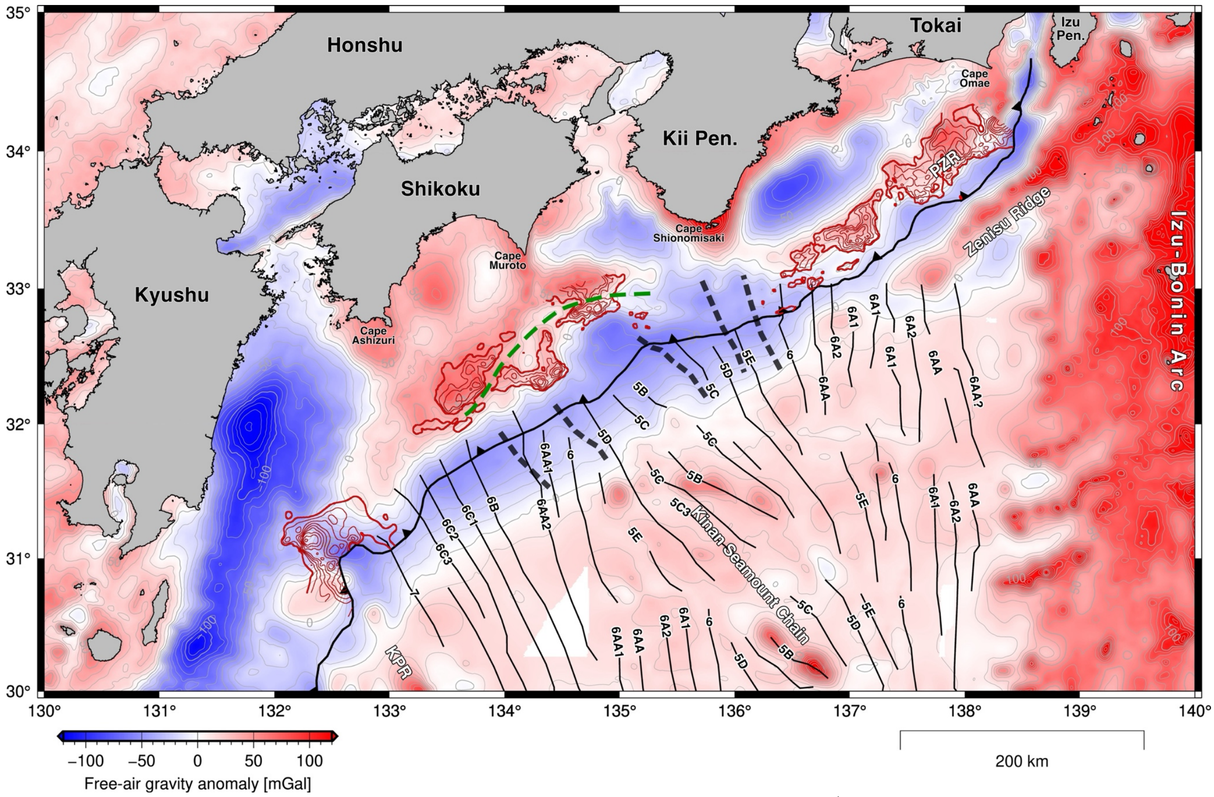

Supplementary Figure S4. Map of the free-air gravity anomaly[1]. The black lines represent the lineation pattern of the magnetic anomaly[2]. The red lines show the contours of the subducting basement surface shallower than 7,000 ms in two-way time every 250 ms (in this study). The black dashed lines represent linear depressions in the north–south direction. The green dashed line shows an arch-shaped valley feature.

References

1. Geological Survey of Japan, AIST. Gravity Database of Japan. DVD edition, Digital Geoscience Map P-2. Geological Survey of Japan, AIST. (2013).

2. Okino K. Magnetic Anomalies in the Philippine Sea: Implications for Regional Tectonics [in Japanese with English abstract]. *J. Geogr. Chigaku Zasshi* **124**, 729–747 (2015).
